# Aldehyde dehydrogenase 1 expression in basal cell carcinoma, actinic keratosis and Bowen’s disease

**DOI:** 10.3892/mco.2013.106

**Published:** 2013-04-18

**Authors:** MITSUAKI ISHIDA, HIDETOSHI OKABE

**Affiliations:** Department of Clinical Laboratory Medicine and Division of Diagnostic Pathology, Shiga University of Medical Science, Otsu, Shiga 520-2192, Japan

**Keywords:** aldehyde dehydrogenase, basal cell carcinoma, actinic keratosis, Bowen’s disease

## Abstract

Aldehyde dehydrogenase (ALDH) is an enzyme responsible for oxidizing aldehydes to carbonic acids. ALDH1 is an isoform that is thought to be a stem cell marker as it is highly expressed in the stem cells of various organs. However, its expression in basal cell carcinoma (BCC), actinic keratosis (AK) and Bowen’s disease (BD) of the skin has not yet been analyzed. Twenty-five consecutive operative cases each of BCC, AK and BD, as well as 10 normal skin tissues were assessed for ALDH1 expression by immunohistochemistry. In normal skin, ALDH1 expression was observed in the suprabasal cells of the follicular infundibulum, inner cells of the outer root sheath and sebocytes. BCC cases (88%) showed no or focal-positive immunoreactivity for ALDH1. Focal immunopositivity for ALDH1 was observed in 44% of AK cases, while the remaining cases were ALDH1-negative. By contrast, diffuse positive immunoreactivity for ALDH1 was observed in 64% of BD cases. Differential expression patterns of ALDH1 in AK and BD may reflect the distinct cells of origin of these two conditions. Moreover, a low ALDH1 expression in BCC may also reflect the possible origin of BCC, the basal cells of the outer root sheath.

## Introduction

Members of the aldehyde dehydrogenase (ALDH) family are NAD(P)-dependent enzymes involved in catalyzing the oxidation of various endogenous and exogenous aldehydes to corresponding carboxylic acids (1) and are classified into 11 families and 4 subfamilies (2). ALDH1 is an isoform that is highly expressed in the stem cells of various lineages including hematopoietic tissues, neural tissues and mammary gland, and has been shown to play an important functional role in stem cells (3).

In normal tissue, strong ALDH1-positive cells are present in the putative epithelial stem/progenitor cell zones located in the breast, colon and stomach (4). Moreover, a high percentage of ALDH1-positive tumor cells are found in various types of cancer, such as ovarian, colon, lung, pancreatic and liver (4). However, analyses of ALDH1 expression in basal cell carcinoma (BCC), actinic keratosis (AK) and Bowen’s disease (BD) of the skin as well as normal skin tissue have not yet been performed. In this study, we assessed ALDH1 expression in BCC, AK and BD by immunohistochemistry and compared the findings with that of normal skin.

## Materials and methods

### Case selection

Twenty-five formalin-fixed and paraffin-embedded tissue specimens each from consecutive operative BCC, AK and BD cases were selected from the archives of our Diagnostic Pathology Division. The 25 cases of BCC included 16 males and 9 females with an average age of 70.2 years (range, 41–92 years). The 25 AK cases comprised 14 males and 8 females with an average age of 76.6 years (range, 46–95 years). These cases included patients with 2 or 3 lesions. The 25 cases of BD included 12 males and 13 females with an average age of 76.7 years (range, 63–89 years). This study was approved by the ethics committee of Shiga University of Medical Science (Shiga, Japan). All patients gave their consent to participate in this study.

The cases were reviewed by diagnostic pathologists to confirm the diagnosis of BCC, AK and BD, and subclassification of BCC was performed according to the World Health Organization Classification of Tumours. Pathology and Genetics of Skin Tumours (5).

ALDH1 expression was also examined in 10 normal skin specimens from the scalp and face.

### Immunohistochemistry

Immunohistochemical stainings were performed using an autostainer (Benchmark XT System; Ventana Medical Systems, Tucson, AZ, USA) according to the manufacturer’s instructions. The primary antibody used was a mouse monoclonal antibody against human ALDH1 (clone 44/ALDH; BD Transduction Laboratories, Franklin Lakes, NJ, USA), as previously described (4,6,7).

### Evaluation of immunoreactivity

The expression pattern of ALDH1 was evaluated semiquantatively as a percentage of positively stained tumor cells, as described in a previous study (8) and scored as: 0 (<5% of positive tumor cells), 1+ (5–9%), 2+ (10–50%) and 3+ (>51%).

## Results

### Normal skin

**Figure 1:**
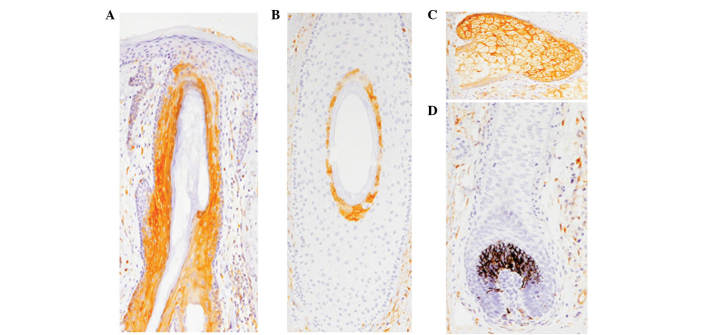
Aldehyde dehydrogenase 1 (ALDH1) expression in normal skin. (A) Suprabasal cells of the follicular infundibulum, (B) the inner cells of the outer root sheath and (C) sebocytes are positive for (D) ALDH1. No positive cells are present in the hair matrix (magnification, ×200).

ALDH1 expression was observed in the suprabasal cells of the follicular infundibulum, mature sebocytes and the inner cells of the outer root sheath (Fig. 1). No ALDH1-positive cells were found in the epidermis, basal cells of the follicular infundibulum or outer root sheath, with the exception of the inner cells, bulge, inner root sheath, hair matrix and supramatrix (Fig. 1).

### BCC

**Figure 2:**
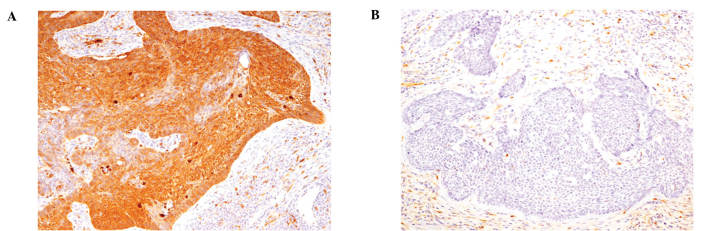
Aldehyde dehydrogenase 1 (ALDH1) expression in basal cell carcinomas. (A) Diffuse positive immunoreactivity for ALDH1. (B) No positive tumor cells are evident (magnification, ×100).

The 25 cases of BCC included 18 nodular types, 4 micronodular types and 3 superficial types. ALDH1 was expressed in 68% of BCC cases. However, only 2 cases showed diffuse positive immunoreactivity (score 3+) (Fig. 2A) and 1 case was scored 2+, whereas 56% of BCC cases showed only focal immunopositivity (score 1+) and 32% were negative (score 0) (Fig. 2B and Table I). Only nodular types of BCC had scores of 2+ or 3+, while the micronodular cases had a score of 1+ and the superficial cases were scored 0 (Table I).

### AK

**Figure 3:**
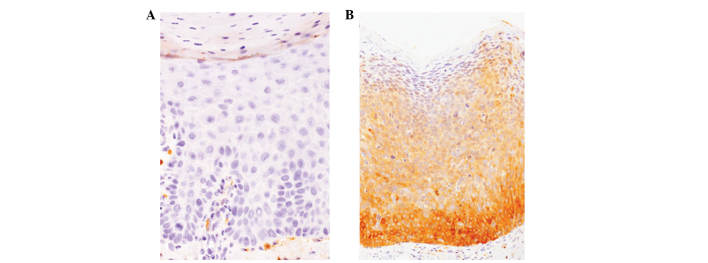
Aldehyde dehydrogenase 1 (ALDH1) expression in actinic keratosis and Bowen’s disease. (A) No positive immunoreactivity for ALDH1 in actinic keratosis. (B) Diffuse positive immunoreactivity in Bowen’s disease (magnification, ×200).

Most of the AK cases (56%) were negative for ALDH1 (score 0) (Fig. 3A) and the remaining 44% of cases had a score of 1+. No diffusely ALDH1-positive cases were observed (Table I).

### BD

ALDH1 expression was observed in 92% of BD cases, of which 64% showed diffuse positive immunoreactivity for ALDH1 (score 3+) (Fig. 3B). Only 8% of BD cases were ALDH1-negative (Table I).

## Discussion

ALDH1 has been proven to be useful for the identification of normal stem cells of various organs. ALDH1-positive tumor cells exhibit cancer stem cell properties and are resistant to chemotherapy in certain types of cancer (3). For example, it has been demonstrated that ALDH1-positive cells have stem or progenitor cell abilities in normal breast and breast cancer cells (8). It has also been shown that the presence of ALDH1-positive tumor cells in lymph node metastatic lesions after neoadjuvant chemotherapy correlated with poor prognosis and reduced survival in breast cancer patients (6). Moreover, a high expression of ALDH1 has been found to be associated with lymph node metastasis in oral squamous cell carcinoma (9) and is also associated with postoperatrive recurrence and poor prognosis in esophageal squamous cell carcinoma (10).

However, the expression of ALDH1 in normal tissues is not always restricted to stem cells. Deng *et al* (4) classified ALDH1 expression patterns in normal tissues into three types: i) tissues with absent or limited ALDH1 expression (breast and lung); ii) tissues with relatively weak ALDH1 expression (stomach and colon); and iii) tissues with extensive and high ALDH1 expression (liver and pancreas). Based on these results, those authors concluded that ALDH1 can be an effective and useful stem cell marker for tissues that usually do not express ALDH1 at a high level, such as breast, stomach and colon. However, ALDH1 should not be used in organs that usually express a high level of ALDH1, such as liver and pancreas (3). To the best of our knowledge, the present study is the first to clearly show that ALDH1 is expressed in the suprabasal cells of the follicular infundibulum, inner cells of the outer root sheath and sebocytes of normal skin tissue. These distribution patterns do not correspond to those of stem cells in the skin, which are thought to be located in the bulge (11). Therefore, ALDH1 is not a useful stem cell marker for normal skin tissue and may have other functional roles as it has been reported that ALDH1 is, not only a putative stem cell marker, but may also have numerous functions, such as in differentiation and self-renewal (3).

This study also clearly demonstrated that over half of AK cases (56%) were negative or negligible for ALDH1. By contrast, 64% of BD cases were diffusely positive for ALDH1. Basal cells of the epidermis are important in the pathogenesis of AK, but not in BD, in which the neoplastic cells have been reported to originate from the pilar outer root sheath or acrotrichium (12). Therefore, the differential ALDH1 expression patterns suggest that in AK and BD, the neoplastic squamous cells harbor distinct phenotypes and may reflect the different origin of these two conditions.

Findings of this study, demonstrated that 88% of BCC cases showed no or only focal-positive immunoreactivity for ALDH1. The possible origin of BCC cells is thought to be the outer root sheath of hair follicles, particularly basal cells (13). ALDH1 expression was observed in the inner cells, but not in the basal cells of the outer root sheath of normal hair follicles. Thus, a low ALDH1 expression in this disease may reflect the possibility that BCC originates from the basal cells of the outer root sheath.

In addition, a recent study demonstrated that high ALDH1-expressing breast cancer cells preferentially survive both chemotherapy and radiation compared with low ALDH1-expressing cancer cells and a specific ALDH inhibitor (diethylaminobenzalaldehyde) may result in significant sensitization to therapy in the former cells (14). Our results have shown that 92% of BD cases showed positive immunoreactivity for ALDH1 (of which 64% showed diffuse expression). Therefore, current administration of an ALDH1 inhibitor might be a candidate treatment for BD.

## Figures and Tables

**Table I:** ALDH1 expression in basal cell carcinoma, actinic keratosis and Bowen’s disease.

	ALDH1 expression			
Carcinoma type	0	1+	2+	3+
Basal cell carcinoma	8/25	14/25	1/25	2/25
Nodular type	5/18	10/18	1/18	2/18
Micronodular type	0/4	4/4	0/4	0/4
Superficial type	3/3	0/3	0/3	0/3
Actinic keratosis	14/25	11/25	0/25	0/25
Bowen’s disease	2/25	5/25	2/25	16/25

0, <5% of positive tumor cells; 1+, 5–9%; 2+, 10–50%; 3+ >51%. ALDH1, aldehyde dehydrogenase 1.

## References

[1] Sladek NE (2003). Human aldehyde dehydrogenases: potential pathological, pharmacological, and toxicological impact. J Biochem Mol Toxicol.

[2] Black WJ, Stagos D, Marchitti SA (2009). Human aldehyde dehydrogenase genes: alternatively spliced transcriptional variants and their suggested nomenclature. Pharmacogenet Genomics.

[3] Ma I, Allan AL (2011). The role of human aldehyde dehydrogenase in normal and cancer stem cells. Stem Cell Rev and Rep.

[4] Deng S, Yang X, Lassus H (2011). Distinct expression levels and patterns of stem cell marker, aldehyde dehydrogenase isoform 1 (ALDH1), in human epithelial cancers. PLoS One.

[5] Kossard S, Epstein EH, Cerio R, Yu LL, Weedon D, LeBoit PE, Burg G, Weedon D, Sarasain A (2006). Basal cell carcinoma. World Health Organization Classification of Tumours. Pathology and Genetics of Skin Tumours.

[6] Sakakibara M, Fujimori T, Miyoshi T (2012). Aldehyde dehydrogenase 1-positive cells in axillary lymph node metastases after chemotherapy as a prognostic factor in patients with lymph node-positive breast cancer. Cancer.

[7] Isfoss BL, Holmqvist B, Alm P, Olsson H (2012). Distribution of aldehyde dehydrogenase 1-positive stem cells in benign mammary tissue from women with and without breast cancer. Histopathology.

[8] Ginestier C, Hur MH, Charafe-Jauffret E (2007). ALDH1 is a marker of normal and malignant human mammary stem cells and a predictor of poor clinical outcome. Cell Stem Cell.

[9] Michifuri Y, Hirohashi Y, Torigoe T (2012). High expression of ALDH1 and SOX2 diffuse staining pattern of oral squamous cell carcinomas correlates to lymph node metastasis. Pathol Int.

[10] Minato T, Yamamoto Y, Seike J (2012). Aldehyde dehydrogenase 1 expression is associated with poor prognosis in patients with esophageal squamous cell carcinoma. Ann Surg Oncol.

[11] Goldstein J, Horsley V (2012). Home sweet home: skin stem cell niches. Cell Mol Life Sci.

[12] Saglam O, Salama M, Meier F (2008). Immunohistochemical staining of palisading basal cells in Bowen’s disease and basal involvement in actinic keratosis: contrasting staining patterns suggest different cells of origin. Am J Dermatopathol.

[13] Ishida M, Kushima R, Okabe H (2008). Immunohistochemical demonstration of D2-40 in basal cell carcinomas of the skin. J Cutan Pathol.

[14] Croker AK, Allan AL (2012). Inhibition of aldehyde dehydrogenase (ALDH) activity reduces chemotherapy and radiation resistance of stem-like ALDHhiCD44+ human breast cancer cells. Breast Cancer Res Treat.

